# Supplementary material for: 90K/Mac-2 BP Is a New Predictive Biomarker of Response to Infliximab Therapy in IBD Patients
Source: Int J Mol Sci. 2023 Feb 16;24(4):3955. doi: 10.3390/ijms24043955 (PMC9966915; doi:10.3390/ijms24043955)
Supplement: Supplementary file 1 [file ijms-24-03955-s001.zip › ijms-2202961-SI.pdf]

Supplementary Materials

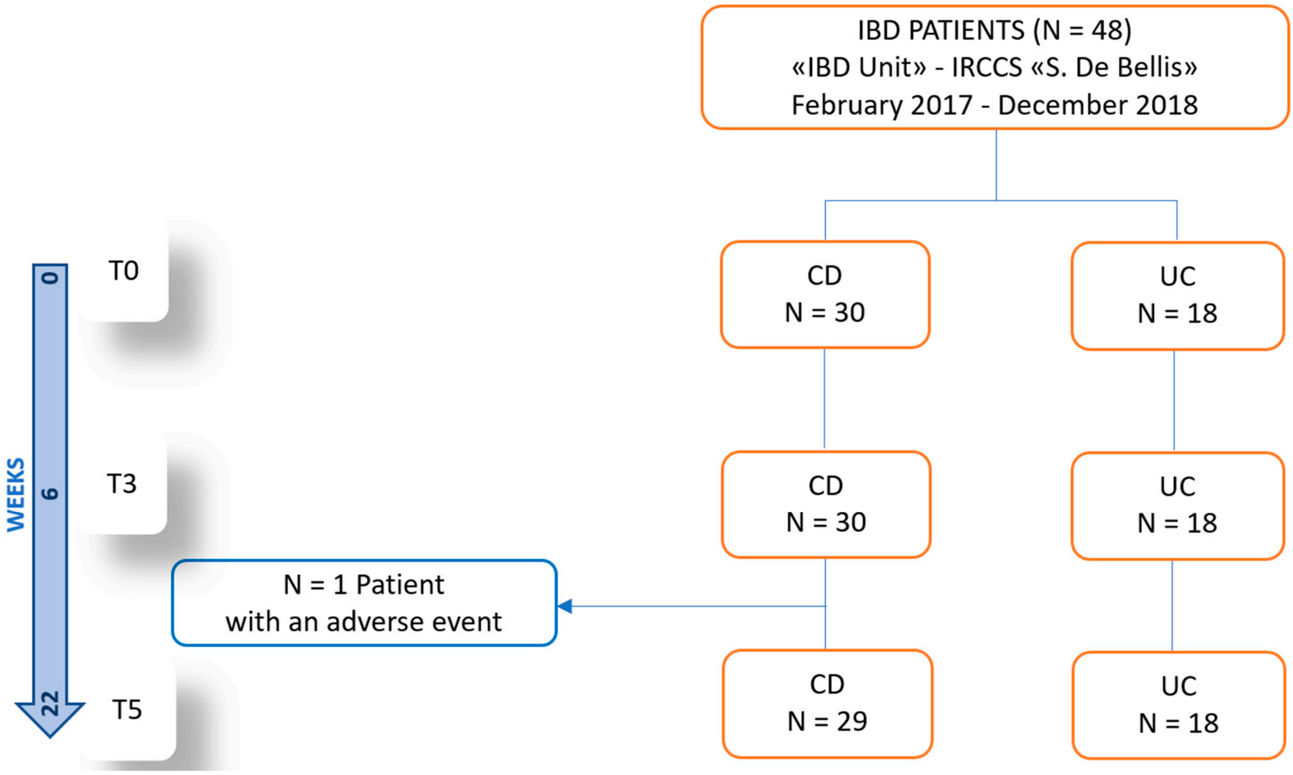

Figure S1. Flowchart showing the selection of the patients, the number of CD and UC, and the dropouts. IBD: inflammatory bowel disease; CD: Crohn's disease; UC: ulcerative colitis.

Table S1. Comparison between infusion cycles and baseline, and trend for the patients with Crohn's disease (N=29). Statistically significant differences (p-value ≤ 0.05) are indicated in bold.

Parameters †	Infusion cycles ‡					
	T0 *(a)*	T3 *(b)*	p # *(b vs a)*	T5 *(c)*	p § *(c vs a)*	p for Trend ¶
25OH-Vitamin D (ng/mL)	24.08±9.72	23.58±9.30	0.84	24.09±9.12	0.54	0.68
90k (µg/mL)	7.78±4.56	8.22±4.27	0.25	8.52±4.20	0.56	0.63
CRP (mg/dL)	1.55±2.31	0.58±1.34	**0.006**	0.54±0.89	**0.0002**	**0.02**
Iron (µg%)	62.82±66.67	75.73±76.82	**0.03**	85.04±47.17	**0.01**	0.28
Ferritin (ng/mL)	64.95±61.96	36.62±37.37	**0.006**	78.42±139.48	0.42	0.69
FCP(mg/kg)	622.34±303.66	240.07±98.10	**<0.0001**	158.30±90.40	**<0.0001**	**<0.0001**

† As Mean and Standard Deviation (M±SD).

‡ Baseline (T0), prior to the 3rd (T3) and 5th (T5) infusion.

§ Wilcoxon signed-rank test; ¶ Test for trend.

CRP: C-reactive protein; FCP: fecal calprotectin.

Table S2. Comparison between infusion cycles and baseline, and trend for the patients with ulcerative colitis (N=18). Statistically significant differences (p-value ≤ 0.05) are indicated in bold.

Parameters †	Infusion cycles ‡					
	T0 *(a)*	T3 *(b)*	p # *(b vs a)*	T5 *(c)*	p § *(c vs a)*	p for Trend ¶
25OH-Vitamin D (ng/mL)	26.70±13.39	27.78±15.70	0.99	22.46±7.68	0.99	0.46
90k (µg/mL)	7.59±3.36	6.46±3.05	0.17	6.22±2.25	0.99	0.28
CRP (mg/dL)	2.01±3.93	0.52±0.95	0.09	0.38±0.57	**0.02**	0.09
Iron (µg%)	46.82±27.17	69.65±35.09	**0.02**	75.42±55.95	0.39	**0.04**
Ferritin (ng/mL)	57.13±51.53	59.64±71.27	0.63	70.40±68.93	0.77	0.60
FCP(mg/kg)	598.88±217.95	301.63±146.49	**0.004**	213.53±189.66	**0.001**	**<0.0001**

† As Mean and Standard Deviation (M±SD).

‡ Baseline (T0), prior to the 3rd (T3) and 5th (T5) infusion.

§ Wilcoxon signed-rank test; ¶ Test for trend.

CRP: C-reactive protein; FCP: fecal calprotectin.

Table S3. Analysis of variance for repeated measures, at Baseline, 3rd and 5th infusion, for single measured parameters in CD patients with circulating ITLs (No/Yes; Number of patients: No=16; Yes=13). Statistically significant differences (p-value ≤ 0.05) are indicated in bold.

Parameters† (CD)	Infusion cycles ‡				Effects p §		Contrasts p ¶ *vs (a)*	
	ITLs	T0 *(a)*	T3 *(b)*	T5 *(c)*	ITLs	Time	*(b)*	*(c)*
90k (μg/mL)	--	7.78±4.56	7.91±4.34	8.32±4.13	0.50	0.66	--	--
	No	8.54±4.58	8.21±3.75	8.78±4.01	--	--	0.65	0.74
	Yes	7.06±4.69	7.57±5.27	7.76±4.38	--	--	0.54	0.40
	p-value ¥	*0.38*						
CRP (mg/dL)	--	1.55±2.31	0.56±1.30	0.49±0.86	0.37	**0.007**	--	--
	No	2.03±2.68	0.41±0.49	0.68±1.11	--	--	**0.001**	**0.02**
	Yes	1.06±1.78	0.73±1.90	0.26±0.25	--	--	0.52	0.11
	p-value ¥	*0.12*						
FCP (mg/kg)	--	622.34±303.66	251.59±92.55	150.62±90.79	0.13	**<0.0001**	--	--
	No	665.00±324.75	257.27±88.40	199.47±99.24	--	--	**<0.001**	**<0.001**
	Yes	569.38±294.27	235.08±95.60	96.15±38.68	--	--	**<0.001**	**<0.001**
	p-value ¥	*0.19*						

† As mean and Standard Deviation (M±SD).

‡ Baseline (T0), prior to the 3rd (T3) and 5th (T5) infusion.

¥ ITLs at 5th (T5) infusion; § ANOVA for repeated measures; ¶ Contrasts of marginal linear predictions.

CD: Crohn's disease; ITLs: infliximab trough levels; CRP: C-reactive protein; FCP: fecal calprotectin.

Table S4. Analysis of variance for repeated measures, at Baseline, 3rd and 5th infusion, for single measured parameters in UC patients with circulating ITLs (No/Yes; Number of patients: No=12; Yes=6). Statistically significant differences (p-value ≤ 0.05) are indicated in bold.

Parameters † (UC)	Infusion cycles ‡				Effects p §		Contrasts p ¶ *vs (a)*	
	ITLs	T0 *(a)*	T3 *(b)*	T5 *(c)*	ITLs	Time	*(b)*	*(c)*
90k (μg/mL)	--	7.59±3.36	7.64±3.88	6.39±2.29	0.65	0.34	--	--
	No	7.60±3.35	7.99±4.56	6.52±2.57	--	--	0.64	0.55
	Yes	7.57±3.69	6.94±2.12	6.15±1.85	--	--	0.58	0.22
	p-value ¥	*0.98*						
CRP (mg/dL)	--	2.01±3.93	0.56±0.97	0.46±0.60	0.73	0.10	--	--
	No	2.34±4.52	0.77±1.19	0.60±0.73	--	--	0.07	**0.05**
	Yes	1.35±2.61	0.22±0.25	0.28±0.31	--	--	0.40	0.40
	p-value ¥	*0.45*						
FCP (mg/kg)	--	598.88±217.95	289.23±162.73	233.12±192.07	0.12	**<0.0001**	--	--
	No	579.73±249.33	342.91±173.47	310.00±199.09	--	--	0.002	<0.001
	Yes	634.00±159.31	190.83±81.23	92.17±43.93	--	--	<0.001	<0.001
	p-value ¥	*0.56*						

† As mean and Standard Deviation (M±SD).

‡ Baseline (T0), prior to the 3rd (T3) and 5th (T5) infusion.

¥ ITLs at 5th (T5) infusion; §ANOVA for repeated measures; ¶ Contrasts of marginal linear predictions.

UC: ulcerative colitis; ITLs: infliximab trough levels; CRP: C-reactive protein; FCP: fecal calprotectin.

Table S5. Analysis of variance for repeated measures, at Baseline, 3rd and 5th infusion, for single measured parameters in the presence of ATIs at T5 (No/Yes) in the patients with Crohn's disease (Number of patients: No=16; Yes=13). Statistically significant differences (p-value ≤ 0.05) are indicated in bold.

**Parameters** † (CD)	**Infusion cycles** ‡				**Effects** p §		**Contrasts p** ¶ *vs (a)*	
	ATIs	T0 *(a)*	T3 *(b)*	T5 *(c)*	ATIs	Time	*(b)*	*(c)*
90k (μg/mL)	--	7.78±4.56	7.91±4.34	8.32±4.13	**0.05**	0.75	--	--
	Yes	10.05±5.42	9.50±4.81	9.45±4.70	--	--	0.49	0.46
	No	6.11±2.95	6.64±3.75	7.40±3.49	--	--	0.46	0.08
	p-value ¥	***0.01***						
CRP (mg/dL)	--	1.55±2.31	0.56±1.30	0.49±0.86	**0.05**	**0.004**	--	--
	Yes	2.46±3.10	0.88±1.92	0.75±1.31	--	--	**0.004**	**0.008**
	No	0.90±1.15	0.28±0.23	0.31±0.22	--	--	0.20	0.22
	p-value ¥	***0.01***						
FCP (mg/kg)	--	622.34±303.66	251.59±92.55	150.62±90.79	0.88	**<0.0001**	--	--
	Yes	610.15±312.83	252.77±100.72	168.69±121.30	--	--	**<0.001**	**<0.001**
	No	629.67±316.49	241.93±84.50	136.60±57.60	--	--	**<0.001**	**<0.001**
	p-value ¥	*0.79*						

† As mean and Standard Deviation (M±SD).

‡ Baseline (T0), prior to the 3rd (T3) and 5th (T5) infusion.

¥ ATIs at 5th (T5) infusion; §ANOVA for repeated measures; ¶ Contrasts of marginal linear predictions.

CD: Crohn's disease; ATIs: antibodies to infliximab; CRP: C-reactive protein; FCP: fecal calprotectin.

Table S6. Analysis of variance for repeated measures, at Baseline, 3rd and 5th infusion, for single measured parameters in the presence of ATIs at T5 (No/Yes) in the patients with UC (Number of patients: No=13; Yes=5). Statistically significant differences (p-value ≤ 0.05) are indicated in bold.

Parameters † (UC)	**Infusion cycles** ‡				**Effects** p §		**Contrasts p** ¶ *vs (a)*	
	ATIs	T0 *(a)*	T3 *(b)*	T5 *(c)*	ATIs	Time	*(b)*	*(c)*
90k (µg/mL)	--	7.59±3.36	7.64±3.88	6.39±2.29	0.09	0.20	--	--
	Yes	9.03±1.66	10.58±5.25	7.03±3.36	--	--	0.20	0.50
	No	7.04±3.73	6.50±2.67	6.19±1.99	--	--	0.47	0.26
	p-value ¥	*0.23*						
CRP (mg/dL)	--	2.01±3.93	0.56±0.97	0.46±0.60	0.98	0.16	--	--
	Yes	1.99±2.12	0.56±0.37	0.67±0.18	--	--	0.31	0.44
	No	2.01±4.52	0.56±1.12	0.42±0.65	--	--	0.10	0.06
	p-value ¥	*0.99*						
FCP (mg/kg)	--	598.88±217.95	289.23±162.73	233.12±192.07	0.49	**0.0001**	--	--
	Yes	569.75±207.43	314.75±73.47	360.00±292.69	--	--	**0.04**	0.09
	No	607.85±228.49	281.38±183.51	194.08±144.05	--	--	**<0.001**	**<0.001**
	p-value ¥	*0.73*						

† As mean and Standard Deviation (M±SD).

‡ Baseline (T0), prior to the 3rd (T3) and 5th (T5) infusion.

¥ ATIs at 5th (T5) infusion; §ANOVA for repeated measures; ¶ Contrasts of marginal linear predictions.

UC: ulcerative colitis; ATIs: antibodies to infliximab; CRP: C-reactive protein; FCP: fecal calprotectin.
